# Amiodarone-Induced Interstitial Pneumonia: A Cause of Respiratory Failure

**DOI:** 10.7759/cureus.74253

**Published:** 2024-11-22

**Authors:** Sofia Eusébio, Ana Raquel Soares, Pedro Fiúza, Teresa Garcia

**Affiliations:** 1 Internal Medicine, Hospital de Santa Marta, Unidade Local de Saúde São José, Lisbon, PRT

**Keywords:** 1:1 atrial flutter, adverse effects, amiodarone, amiodarone pulmonary toxicity, interstitial pneumonitis

## Abstract

Amiodarone, a widely used antiarrhythmic medication, is effective for managing various types of cardiac arrhythmias. However, due to its high lipid solubility and long half-life, amiodarone accumulates in various organs, particularly the lungs. Pulmonary toxicity, while rare (1% to 5% incidence), is among the most serious adverse effects of amiodarone, with interstitial pneumonitis (IP) being the most prevalent form of lung toxicity. Recognized risk factors are old age, high daily doses, and long-lasting therapy. Amiodarone-induced interstitial pneumonia (AIP) is a rare but serious complication of amiodarone therapy, often presenting as progressive respiratory failure. Patients with AIP typically present with nonspecific respiratory symptoms, including dyspnea, cough, and occasionally fever, making the initial diagnosis challenging. Diagnosing AIP requires a combination of clinical, radiological, and histological data, with computed tomography (CT) often revealing ground-glass opacities and interstitial thickening. Management of AIP focuses on discontinuing amiodarone and initiating corticosteroid therapy. Early withdrawal of amiodarone is crucial for symptom resolution and preventing progression to severe lung injury or irreversible fibrosis.

We report an 89-year-old female patient with chronic use of amiodarone who was medicated with high doses of the drug during hospitalization and started presenting signs of respiratory failure. The radiological findings were consistent with AIP, and the patient improved with drug discontinuation and a trial of glucocorticoid therapy. AIP is a critical but preventable cause of respiratory failure in patients undergoing antiarrhythmic therapy. Awareness among healthcare providers about this complication, along with timely diagnosis and management, is essential to improving patient outcomes.

## Introduction

Atrial fibrillation (AF) is the most common arrhythmia needing treatment, with its occurrence doubling every decade starting from the age of 55. Amiodarone, a benzofuran derivative that contains iodine, is classified as a class III antiarrhythmic drug and is effective for both atrial and ventricular arrhythmias due to its combined β-blocking, calcium channel blocking, and class III antiarrhythmic properties. In cases of persistent AF, studies have shown that amiodarone is successful in achieving and maintaining a normal heart rhythm (50-70% success rate) [1]. However, as the usage of amiodarone has increased, there have been reports of toxicity affecting various organs, including the lungs, liver, heart, thyroid gland, eyes, skin, and nervous system [2].

The toxicity is believed to be due to the deposition of iodine compounds in various organs [1]. The overall occurrence of adverse effects ranges from 30% to 90%, while serious side effects affect between 10% and 26% of patients. Careful monitoring is crucial for patients on amiodarone. Adverse effects are frequent, occurring in up to 15% of patients within the first year and rising to 50% with long-term use. Particularly in cases of non-life-threatening arrhythmias like AF, the risk may outweigh the benefits if side effects develop [3].

Pulmonary toxicity, a potentially fatal side effect of amiodarone, has a mortality rate ranging from 1% to 33%, with an incidence rate of approximately 10%, as reported in previous studies. Patients suffering from amiodarone-induced pulmonary toxicity often exhibit symptoms such as shortness of breath, dry cough, general discomfort, fever, and sharp chest pain. Symptoms are often nonspecific, complicating diagnosis, with imaging commonly revealing ground-glass opacities. Management involves discontinuing amiodarone and initiating corticosteroids, with prompt withdrawal being crucial for recovery. A meta-analysis of 6500 patients concluded that the risk of pulmonary toxicity is ~2%, more common in older patients, with higher doses, and with a longer duration of therapy. Although some studies suggest that pre-existing lung disease is associated with a higher risk of pulmonary toxicity, the cumulative data on this issue is discordant. The most common form of pulmonary toxicity is interstitial pneumonia (IP) in 1% to 5% of patients, which leads to acute respiratory distress requiring mechanical ventilation and has a mortality rate ranging from 50% to 100% [2,4].

This article reports a female elderly patient who developed respiratory failure due to high-dose amiodarone and improved after stopping the medication and starting glucocorticoid therapy.

## Case presentation

A non-smoker 89-year-old female with a history of heart failure and atrial flutter medicated with amiodarone (200mg daily) for two years ago presented to the emergency department with a three-day history of dyspnea and palpitations.

**Figure 1:**
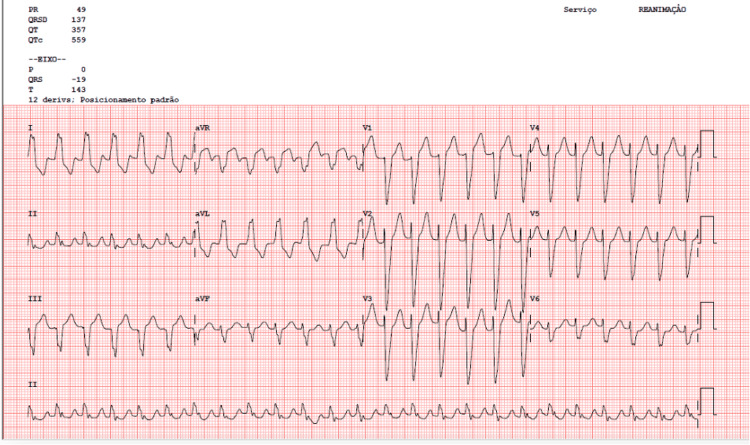
Electrocardiogram showing an atrial flutter (blockage 2:1) with rapid ventricular rate.

On physical examination, the patient exhibited tachycardia (heart rate 150 bpm) and clinical signs of fluid overload (dyspnea and lower extremities bilateral edema). An electrocardiogram revealed atrial flutter with a rapid ventricular rate (Figure 1). After stabilization with intravenous amiodarone (load dose and maintenance), she was admitted to our internal medicine ward with the main diagnosis of heart failure decompensated by atrial flutter with rapid ventricular rate.

In the initial days of hospitalization, the patient experienced several episodes of symptomatic atrial flutter with rapid ventricular rate, prompting the reintroduction of amiodarone, first in a loading dose followed by an infusion over 36 hours. After a discussion with the attending cardiologist, the patient was maintained on oral amiodarone at 200mg twice daily. Regarding congestive heart failure, a cardiorenal syndrome with refractory congestion to high-dose diuretic therapy and concomitant severe acute kidney injury with low urine output was observed. Analytically, there was a progressive increase in inflammatory parameters, but culture tests and virology screenings were negative.

**Figure 2:**
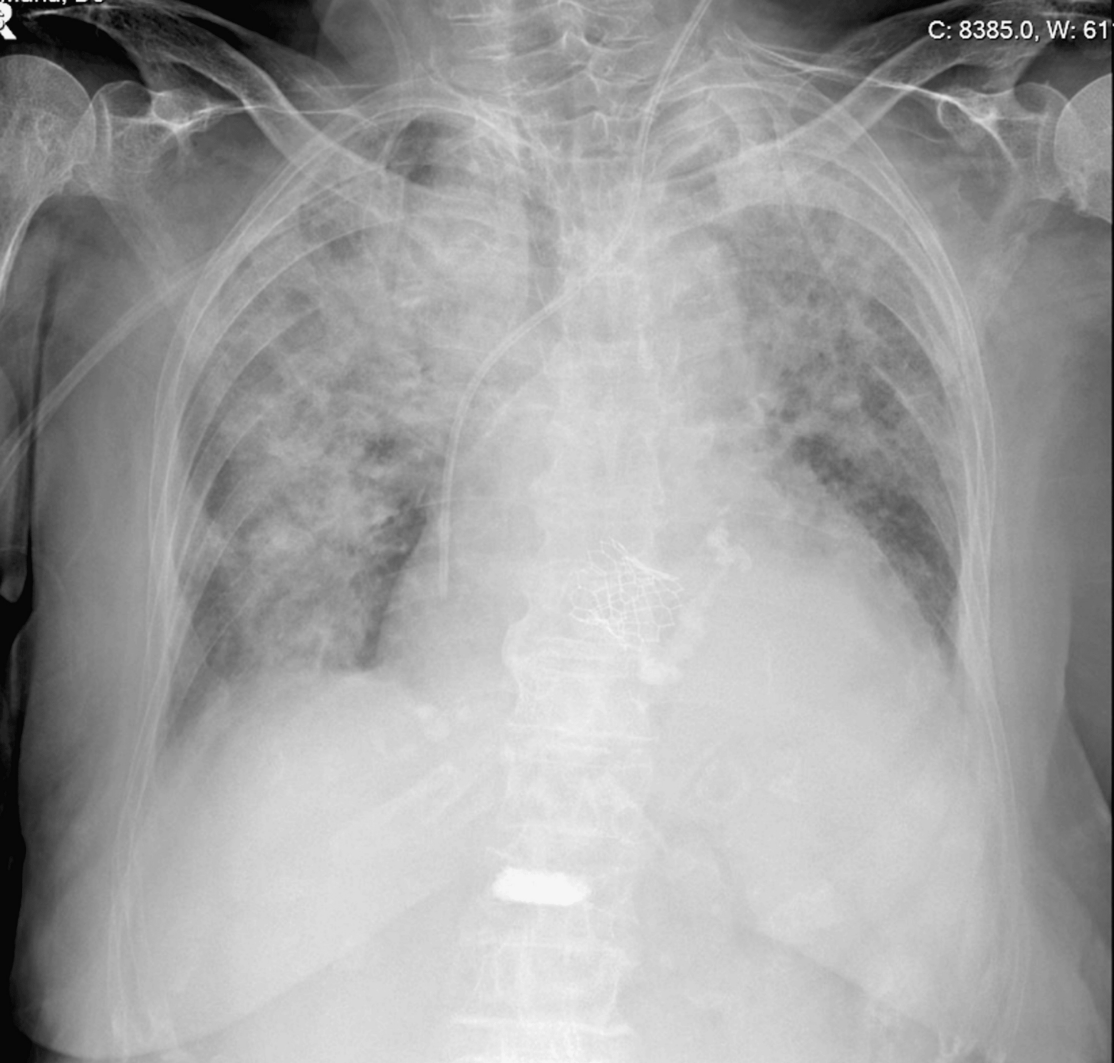
Chest radiograph showing bilateral interstitial infiltrates.

**Figure 3:**
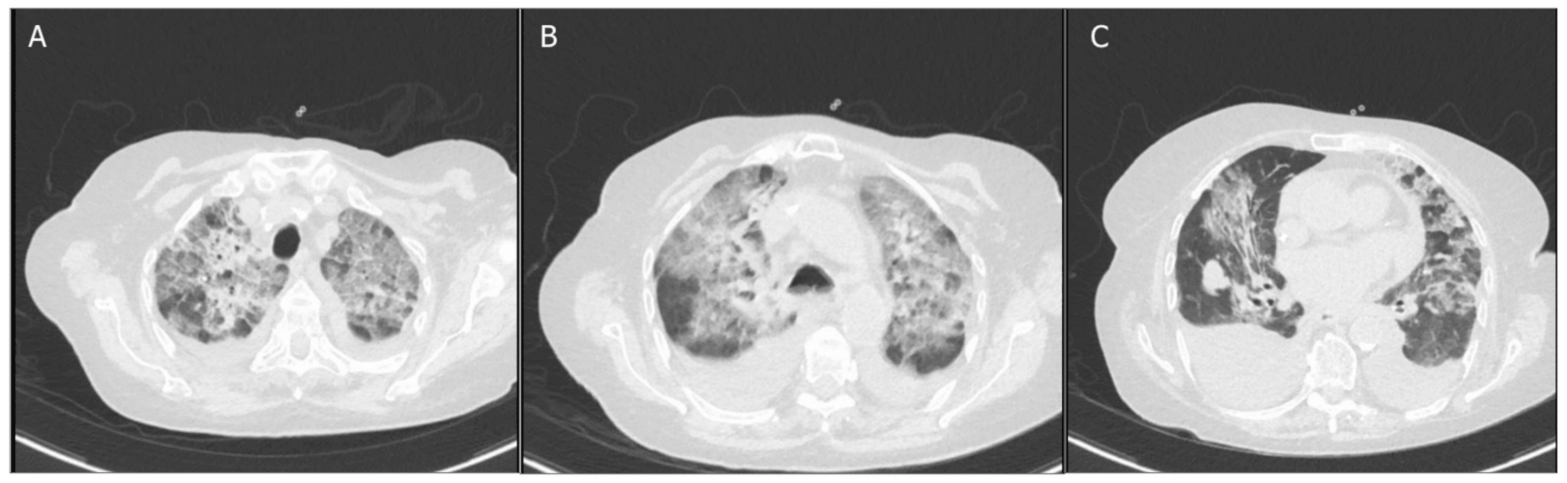
(A-C) Chest computed tomography images showing bilateral pleural effusion and areas with ground-glass opacities and a crazy-paving pattern.

On the eighth day of hospitalization, due to clinical worsening, a follow-up chest radiograph was performed showing new interstitial infiltrates (Figure 2). For further investigation, we performed a chest computed tomography (CT) that demonstrated bilateral pleural effusion at the dependent areas, of moderate volume, causing passive and partial collapse of the lower lobes. Ground-glass opacities and crazy-paving patterns are predominantly observed in the upper lobes, with smaller similar foci in other lobes (Figure 3).

After discussing the imaging findings, clinical evolution, and therapeutic management with the pulmonology colleagues, the most probable diagnosis was assumed to be amiodarone-induced interstitial pneumonia (AIP). Therefore, it was decided to discontinue the drug and introduce prednisolone at 60mg/day, which resulted in a progressive improvement in respiratory failure. However, there was a concurrent worsening of the cardiorenal syndrome with a significant analytical increase in urea and creatinine, decreased urine output, and more evident fatigue at rest. Considering the patient's dependency level and multiple comorbidities, it was decided by the team that invasive resuscitation measures would not be indicated, and on the 12th day of hospitalization, the described clinical deterioration culminated in death.

## Discussion

Amiodarone is commonly used to treat cardiac arrhythmias, but it can sometimes cause side effects affecting various organs. Pulmonary toxicity, although less common than thyroid and eye issues, poses a significant risk and can lead to respiratory failure or even death [5]. IP is the most common form of lung toxicity; it is characterized by new or worsening dyspnea and dry cough on a patient taking ≥200 mg of amiodarone a day, particularly 6 to 12 months into therapy [4]. Diagnosing AIP requires a combination of clinical, radiological, and histological data and drug cessation response. Pulmonary imaging is essential, and the chest CT reveals ground-glass opacities, localized or diffuse parenchymal infiltrates mono- or bilateral, parenchymal nodules, pneumonia, pleural thickening and effusions. The main radiology findings are ground glass and reticular opacities [5]. Pulmonary function tests typically demonstrate a restrictive pattern with reduced diffusing capacity for carbon monoxide, aiding in the assessment of disease severity. Routine lung biopsy in suspected cases is not recommended since the pathologic findings of IP are non-specific. Bronchoscopy with bronchoalveolar lavage, with or without trans-bronchial lung biopsy, may be useful in select cases of suspected pulmonary toxicity. Treatment involves discontinuing amiodarone and, in severe cases, administering corticosteroids. There is a lack of reliable data to determine the appropriate dosing and duration of corticosteroid therapy. Generally, a daily dose of 40-60 mg of prednisone (or an equivalent) is prescribed, often resulting in a rapid response. Although mortality from amiodarone-induced pulmonary toxicity has been reported to reach around 10%, this figure likely reflects the most severely ill patients. Consequently, the actual mortality rate is probably much lower, particularly when the diagnosis is made early [3].

This article presents a case of a patient with chronic use of amiodarone who was medicated with high doses of the drug during hospitalization and started presenting signs of respiratory failure. The radiological findings were consistent with AIP, and the patient improved with drug discontinuation and a trial of glucocorticoid therapy.

Considering the patient's dependency level and multiple comorbidities, it was decided by the team not to perform further investigation and the diagnosis was made by the clinical history, symptoms, radiological findings and treatment response.

## Conclusions

The presented case aims to highlight the need for cautious chronic use of amiodarone, especially in the elderly population with pre-existing pulmonary conditions, due to its high potential for toxicity. Guidelines for monitoring adverse events in patients on long-term amiodarone therapy need to be established. Upon diagnosis, amiodarone should be promptly discontinued, and the use of systemic steroids should be evaluated. It is important to administer the minimum effective dose, remain vigilant for respiratory complaints, and conduct periodic radiological monitoring. Although pulmonary toxicity is less common, it can be fatal. Therefore, timely recognition is essential for a good therapeutic response.
